# Correction to “Energy‐Based Devices in the Treatment of Acne Scars in Skin of Color”

**DOI:** 10.1111/jocd.70174

**Published:** 2025-05-09

Teymour S, Kania B, Lal K, Goldberg D. Energy‐based devices in the treatment of acne scars in skin of color. *J Cosmet Dermatol*. 2023;22(4):1177‐1184.

On page 1179 when referring to reference 10 (Chen et al.), the treatment depths of 60 mm, 200 mm, and 300 mm are incorrect and should be 60 μm, 200 μm, and 300 μm.

We apologize for this error.

